# Fluctuating Star Ratings and Medicare Advantage Bonuses

**DOI:** 10.1001/jamahealthforum.2025.4398

**Published:** 2025-10-24

**Authors:** Andrew Anderson, Mark K. Meiselbach

**Affiliations:** 1 Department of Health Policy and Management, Johns Hopkins Bloomberg School of Public Health, Baltimore, Maryland

## Abstract

This study examines whether Medicare contracts that repeatedly crossed the bonus-eligible threshold were more likely to receive bonus payments.

## Introduction

Medicare Advantage (MA) star ratings determine billions in federal bonuses and shape enrollment, yet it remains unclear whether they reward sustained quality or episodic gains around key thresholds.^1^ Prior research has shown that low star ratings deter enrollment more strongly than high ratings attract it, heightening the stakes for health plans seeking to maintain a favorable public image.^2^ In 2024, quality bonus payments exceeded $11.8 billion, reflecting a major federal investment in performance-based incentives.^3^ Because true quality is expected to remain relatively stable,^4^ volatility-driven bonuses may indicate misaligned incentives. We examined whether contracts that repeatedly crossed the bonus-eligible threshold were more likely to receive bonus payments.

## Methods

We conducted a retrospective analysis of MA contract star ratings from 2015 to 2025 using US Centers for Medicare & Medicaid Services star ratings and contract and enrollment data. Contracts with at least 3 years of data were included. The primary exposure was the number of times a contract entered or exited bonus eligibility (≥4.0 stars). We also estimated models that separated entries and exits to assess directional effects. Outcomes included (1) the proportion of years rated 4.0 or greater, (2) the proportion of consecutive years rated 4.0 or greater, (3) the proportion of years rated 4.0 or greater among ever-eligible contracts, and (4) analogous outcomes for 5-star ratings. We fit linear regression models with robust standard errors, adjusting for mean star rating, log enrollment, average special needs plan, and low-income subsidy enrollment, years observed, and organization type (eg, local coordinated care plan, regional coordinated care plan, private fee for service, medical savings account, employer/cost). Sensitivity analyses used alternative thresholds requiring 2, 4, or 5 years of data. Analyses were performed using Stata/MP 18 (Stata Corp). This study followed Strengthening the Reporting of Observational Studies in Epidemiology (STROBE) reporting guidelines and did not involve human participants; therefore, institutional review board approval was waived.

## Results

Among 552 MA contracts (Table 1), the mean (SD) star rating was 3.67 (0.51), average years observed was 8, and average enrollment was 51 006. Contracts were rated 4.0 stars or greater in 42% of the years observed, and 74% reached that threshold at least once. Among those ever eligible, the mean (SD) share of bonus-eligible years was 58% (0.29%). Contracts crossed the 4-star threshold a mean of 1.6 times, split between 0.80 bonus entries and 0.81 exits. Only 27% of the years observed were part of a consecutive bonus streak, and just 5% were rated 5 stars. Each bonus entry (Table 2) was associated with a 2.3-point increase in the share of years rated 4.0 or greater, but a 3.2-point decrease in consecutive bonus years. Exits were associated with a 6.6-point drop in consecutive bonus years but not overall eligibility. Bonus entries also predicted fewer 5-star years (–3.0 points) and consecutive 5-star years (–1.5 points). Results held across alternative samples (eTables 2-4 in Supplement 1).

**Table 1.** Descriptive Characteristics of 552 Medicare Advantage Contracts From 2015 to 2025^a^

Characteristic	Mean (SD)	Median (range)
Mean star rating^b^	3.67 (0.51)	3.67 (2.33 to 4.91)
Mean enrollment^c^	51 006.06 (131 487.46)	16 570.94 (643.00 to 1 458 967.50)
Log of mean enrollment^d^	9.74 (1.45)	9.72 (6.47 to 14.19)
Mean SNP enrollment^e^	0.18 (0.31)	0.02 (0 to 1.00)
Mean LIS enrollment^e^	0.36 (0.31)	0.23 (0.00 to 1.00)
Years observed^f^	8.04 (3.18)	9.00 (3.00 to 11.00)
Total transitions^g^	1.61 (1.70)	1.00 (0 to 7.00)
Entries into bonus^h^	0.80 (0.87)	1.00 (0 to 3.00)
Exits from bonus^h^	0.81 (0.91)	1.00 (0 to 4.00)
Net transitions^i^	−0.02 (0.54)	0 (−1.00 to 1.00)
Proportion years rated ≥4.0^j^	0.42 (0.36)	0.37 (0.00 to 1.00)
Proportion of years rated ≥4 stars (conditional on ever eligible)^j^	0.58 (0.29)	0.64 (0.09 to 1.00)
Consecutive years rated ≥4.0	0.27 (0.31)	0.14 (0 to 0.91)
Proportion of years rated 5 stars^k^	0.05 (0.13)	0 (0 to 0.82)
Consecutive years rated 5 stars^k^	0.02 (0.07)	0 (0 to 0.64)

Abbreviations: LIS, low-income subsidy; SNP, special needs plan.

^a^ Data source: US Centers for Medicare & Medicaid Services Medicare Advantage star ratings data tables, 2015 to 2025.

^b^ Mean overall rating for each contract across all observed years.

^c^ Mean annual number of enrollees per contract.

^d^ Natural logarithm of total enrollment summed over all years.

^e^ Mean proportion of enrollees in special needs plans and receiving low-income subsidies, respectively.

^f^ Number of years the contract appears in the data (up to 11 years).

^g^ Sum of bonus entries and exits.

^h^ Number of years a contract newly entered or exited bonus eligibility (defined as receiving ≥4 stars).

^i^ Difference between them.

^j^ Share of years with bonus ratings and the share of consecutive bonus years, respectively.

^k^ Fequency and persistence of perfect 5-star ratings over time.

**Table 2.** Association Between Threshold Crossings and Medicare Advantage Bonus Eligibility Outcomes ^a^^,^^b^

Characteristic	Proportion years rated ≥4.0	Consecutive years rated ≥4.0	Proportion of years rated 5 stars	Consecutive years rated 5 stars
Bonus entries	0.023 (0.009)^c^	−0.032 (0.010)^c^	−0.030 (0.006)^c^	−0.015 (0.004)^c^
Bonus exits	−0.013 (0.008)	−0.066 (0.009)^c^	−0.016 (0.006)^c^	−0.009 (0.003)^c^
Mean star rating	0.654 (0.016)^c^	0.543 (0.016)^c^	0.172 (0.015)^c^	0.074 (0.011)^c^
Log total enrollment	0.010 (0.004)^d^	0.008 (0.004)^e^	−0.011 (0.003)^c^	−0.004 (0.002)^d^
Mean (SD) SNP enrollment, %	0.010 (0.034)	0.018 (0.034)	−0.030 (0.022)	−0.026 (0.015)^e^
Mean (SD) LIS enrollment, %	−0.000 (0.000)	−0.001 (0.000)^e^	0.000 (0.000)	0.000 (0.000)
Years observed	−0.002 (0.002)	0.017 (0.002)^c^	0.003 (0.001)^e^	0.002 (0.001)^c^
Observations	512.000	512.000	512.000	512.000
*R* ^2^	0.884	0.852	0.527	0.345

Abbreviations: LIS, low-income subsidy; SNP, special needs plan.

^a^ Data source: Centers for Medicare & Medicaid Services Medicare Advantage star ratings data tables, 2015 to 2025.

^b^ Linear regression models estimate associations between bonus threshold transitions and star rating stability outcomes. Columns 1 and 2 examine the proportion of years rated ≥4 stars and consecutive years rated ≥4 stars; columns 3 and 4 examine the proportion and consecutive years rated 5 stars. Independent variables include the number of bonus entries and exits, mean overall star rating, log total enrollment, average SNP enrollment percentage, average LIS enrollment percentage, years observed, and plan type. The models also adjust for variation in organizations types within contracts (not shown), including local coordinated care plans, regional coordinated care plans, private fee-for-service plans, and medical savings account plans. The reference group is 1876 cost plans. Robust standard errors are shown in parentheses.

^c^ *P* < .01.

^d^ *P* < .05.

^e^ *P* < .10.

## Discussion

In this study, contracts that frequently crossed the 4-star bonus threshold were more likely to receive quality bonuses despite only modest differences in average performance. These findings suggest the current bonus structure may reward volatile, threshold-sensitive performance over consistent quality. Threshold crossings were associated with not only ever receiving a bonus, but also with a higher share of bonus-eligible years, suggesting that episodic gains may repeatedly cross key thresholds. These movements may reflect actual changes in quality or instability in management, reporting, or patient experience, but their association with bonus eligibility raises concerns about whether incentives support sustained performance improvement.^5^ Limitations included potential selection bias from restricting to contracts with at least 3 years of data and the absence of controls for regional and some organizational variation. However, threshold crossings were consistently measured across contracts, and associations were robust across model specifications. These findings raise concerns about whether star ratings capture sustained excellence or disproportionately reward performance volatility that may not reflect true quality.

## Notes

Supplement 1. eMethods. eFigure. Distribution of Medicare Advantage Star Ratings, 2015-2025 eTable 1. Direction of Annual Star Rating Change by Year (Contract-Year Observations) eTable 2. Robustness of Bonus Eligibility Models With 2-Year Minimum Observation Requirement eTable 3. Robustness of Bonus Eligibility Models With 4-Year Minimum Observation Requirement eTable 4. Robustness of Bonus Eligibility Models With 5-Year Minimum Observation Requirement eTable 5. Volatility and Bonus Eligibility by Parent Organization

Supplement 2. Data sharing statement
