# Supplementary material for: Fluctuating Star Ratings and Medicare Advantage Bonuses
Source: JAMA Health Forum. 2025 Oct 24;6(10):e254398. doi: 10.1001/jamahealthforum.2025.4398 (PMC12552924; doi:10.1001/jamahealthforum.2025.4398)
Supplement: Supplement 1. — eMethods. eFigure. Distribution of Medicare Advantage Star Ratings, 2015-2025 eTable 1. Direction of Annual Star Rating Change by Year (Contract-Year Observations) eTable 2. Robustness of Bonus Eligibility Models With 2-Year Minimum Observation Requirement eTable 3. Robustness of Bonus Eligibility Models With 4-Year Minimum Observation Requirement eTable 4. Robustness of Bonus Eligibility Models With 5-Year Minimum Observation Requirement eTable 5. Volatility and Bonus Eligibility by Parent Organization [file jamahealthforum-e254398-s001.pdf]

Supplemental Online Content

Anderson A, Meiselbach MK. Fluctuating star ratings and Medicare Advantage bonuses. *JAMA Health Forum*. 2025;6(1):e254398. doi:10.1001/jamahealthforum.2025.4398

eMethods.

eFigure. Distribution of Medicare Advantage Star Ratings, 2015-2025

eTable 1. Direction of Annual Star Rating Change by Year (Contract-Year Observations)

eTable 2. Robustness of Bonus Eligibility Models With 2-Year Minimum Observation Requirement

eTable 3. Robustness of Bonus Eligibility Models With 4-Year Minimum Observation Requirement

eTable 4. Robustness of Bonus Eligibility Models With 5-Year Minimum Observation Requirement

eTable 5. Volatility and Bonus Eligibility by Parent Organization

This supplemental material has been provided by the authors to give readers additional information about their work.

eMethods.

Dataset Construction

We constructed a contract-year–level dataset by merging CMS files on Medicare Advantage (MA) Star Ratings and Part D Contract and Enrollment Data from 2015 to 2025. Measurement years reflect the prior year's performance (e.g., 2025 measurement year reflects 2024). For each source, we harmonized variable names and formats across years, using contract ID, year, and plan benefit package (PBP) as the unit of alignment. We extracted overall star ratings from CMS Star Rating data tables, aggregated monthly enrollment to annual totals, and calculated LIS share based on the ratio of LIS to total enrollment within each contract-year. Missing values in the proportion of enrollees receiving the LIS were filled using values from adjacent years within the same contract. If still missing, we assigned the contract-level mean LIS share. After merging all panels, we collapsed the dataset to the contract-year level, aggregating PBPs to produce the analytic file used in this study.

Unit of Analysis

The unit of analysis is the MA contract-year, representing all plans grouped under a single CMS contract identifier in a given year. In the MA program, a contract reflects the formal agreement between a private insurer and the CMS and may include multiple PBPs offering distinct benefit structures (e.g., HMO vs. PPO). Because star ratings are assigned at the contract—not plan—level, our analysis aggregates performance accordingly. We limited the sample to contracts with at least three years of star rating data between 2015 and 2025 to enable the assessment of longitudinal variation. The final analytic sample includes 552 contracts, followed for at least 3 years and over 5,000 contract-year observations.

Star Rating Thresholds and Bonus Eligibility

CMS provides quality bonus payments to MA contracts that achieve an overall star rating of 4.0 or higher on a 5-star scale. Contracts rated between 4.0 and 4.5 stars receive the standard bonus, while those earning a 5.0-star rating are eligible for enhanced bonuses and year-round enrollment privileges. Although star ratings are reported in 0.5-point increments (e.g., 3.0, 3.5, 4.0, 4.5), the threshold of ≥4.0 is the primary policy-relevant cutoff tied to financial incentives. In this study, we define bonus eligibility as receiving a rating of 4.0 or higher in a given year. While 5.0-star contracts are eligible for higher rebate percentages, our analysis centers on entry into and the stability of standard bonus eligibility (≥4.0), which applies to the majority of contracts and accounts for most bonus payments.

Primary Exposure Variables

To assess how fluctuations in performance affect bonus eligibility, we focused on contract-level transitions across the 4.0-star threshold—the level used by CMS to determine quality bonus payments. The main exposure variable was the total number of threshold crossings, defined as the sum of all entries into and exits from bonus eligibility over the study period. We also examined directional transitions separately: entries were defined as years in which a contract newly achieved a rating of ≥4.0 after being below that threshold in the prior year, while exits were defined as years in which a contract dropped below 4.0 after qualifying. We also included the proportion of bonus eligible years conditional on having previously achieved 4 stars. These

© 2025 Anderson A et al. *JAMA Health Forum*.

measures capture not only general volatility in performance but also reflect movement into and out of the bonus-eligible tier that triggers financial incentives.

Outcome Measures

We examined four contract-level outcomes to assess the frequency and stability of high performance under the Medicare Advantage Star Ratings system. Two outcomes focused on the policy-relevant 4.0-star threshold: the proportion of years a contract received a rating of ≥4.0 stars, and the proportion of consecutive years rated ≥4.0. These measures reflect both the consistency and durability of bonus eligibility over time. To capture exceptional performance, we also examined the proportion of years a contract was rated 5 stars and the share of consecutive years with 5-star ratings. While relatively few contracts achieve a perfect rating, these outcomes allow us to assess the persistence of peak performance across years. Together, these four measures provide a comprehensive picture of bonus eligibility and performance stability in the Star Ratings program.

Covariates

All models adjusted for contract-level characteristics that could influence star rating performance or volatility. These included the average overall star rating across observed years, the log of total enrollment (as a proxy for contract size), the average percentage of enrollees in Special Needs Plans (SNPs), and the average percentage of enrollees receiving Low-Income Subsidies (LIS). To account for variation in follow-up time, we included the number of years each contract was observed as a covariate. We also adjusted for plan type using categorical indicators for contract classification—such as Local Coordinated Care Plans (Local CCPs), Regional CCPs, Private Fee-for-Service (PFFS) plans, Medical Savings Account (MSA) plans, and Employer/Cost plans. Local CCPs, typically HMOs or PPOs, operate within specific counties and offer uniform benefits across their service area. Regional CCPs span larger geographic areas and are required to include unified deductibles and catastrophic coverage. PFFS plans allow beneficiaries to see any Medicare-approved provider willing to accept the plan's terms, offering greater provider choice but less predictability. MSA plans pair high-deductible coverage with a Medicare-funded savings account for qualified medical expenses. Employer/Cost plans include Employer Group Waiver Plans (EGWPs) sponsored by employers or unions and traditional Medicare Cost plans, which allow beneficiaries to seek care outside the plan's network with reimbursement under traditional Medicare rules.

Statistical Analysis

We used linear regression models at the MA contract level to assess associations between bonus threshold crossings and four outcomes: (1) whether a contract ever received a rating ≥4.0 stars, (2) the proportion of observed years rated ≥4.0, (3) the proportion of years rated 5 stars, and (4) the proportion of consecutive years rated 5 stars. All models used ordinary least squares with robust standard errors. Primary exposures included the total number of threshold crossings (entries and exits combined), and in separate models, the number of entries and exits modeled directionally. Models adjusted for mean star rating, the natural log of total enrollment, average SNP enrollment, average low-income subsidy LIS enrollment, years observed, and contract type. Plan type was modeled categorically. All analyses were conducted in Stata/MP 18.

© 2025 Anderson A et al. *JAMA Health Forum*.

Sensitivity Analysis
To evaluate the robustness of our findings to alternative inclusion follow-up periods, we repeated all regression models using progressively stricter sample restrictions—limiting the analytic cohort to contracts with at least 2 years (Appendix Table 2), 4 years (Appendix Table 3), or 5 years (Appendix Table 4) of star rating data. Across all specifications, the direction and statistical significance of key associations remained consistent with the main analysis. In all models, bonus entries were positively associated with the proportion of years rated ≥4.0 and negatively associated with the proportion of 5-star years. The coefficient for bonus entries on the proportion of bonus-eligible years remained stable around 0.002 across specifications, while exits from bonus eligibility were negatively associated with both consecutive bonus years and consecutive 5-star years. Mean star rating was consistently a strong predictor across all outcomes, and covariates such as total enrollment, SNP/LIS enrollment, and plan type showed expected variation in magnitude but not direction. These results show that the relationship between bonus threshold volatility and both bonus attainment and consistency is robust to variation in the minimum number of years observed. While estimates for some covariates changed slightly due to sample composition shifts, the core findings held under each restriction.

Bonus Payment Impact Estimation

To assess the potential financial implications of performance volatility, we used regression-estimated marginal effects from our main analysis (Table 2, Column 1). Specifically, the model estimated that each additional entry into bonus eligibility—defined as crossing the 4.0-star threshold from below—was associated with a 2.3 percentage point increase in the likelihood that a contract received a quality bonus at least once during the study period.

To translate this effect into dollars, we multiplied the marginal effect (2.3 percentage points) by the average number of bonus entries per contract (0.80), the average contract enrollment (51,000 beneficiaries), and an assumed $345 bonus per enrollee, consistent with recent Kaiser Family Foundation (KFF) estimates of average Medicare Advantage bonus payments per enrollee. This yields an estimated $323,748 in bonus payments per contract attributable to marginal increases in bonus eligibility.

Across the full sample of 512 Medicare Advantage contracts, this amounts to an estimated $165.8 million in total bonus payments associated with threshold crossings. This estimate is illustrative and conservative. It assumes one year of bonus payment per marginally affected contract and does not account for variation in enrollment across years, enhanced payments for 5-star plans, or CMS rules related to bonus rounding, hold-harmless provisions, and rebate percentages. We also assume a constant marginal effect across contracts and time. Nonetheless, the findings suggest that even modest rating volatility near the 4-star threshold can produce substantial fiscal consequences for Medicare spending.

- Reference**:** Biniek JF, Freed M, Damico A. Medicare Advantage Quality Bonus Payments Will Total at Least $11.8 Billion in 2024. Kaiser Family Foundation. Published September 11, 2024. Accessed [insert date]. https://www.kff.org/medicare/issue-brief/medicare-advantage-quality-bonus-payments-will-total-at-least-11-8-billion-in-2024/

Volatility and Bonus Eligibility by Parent Organization: Appendix Table 5 highlights variation in bonus eligibility volatility across major MA parent organizations. United Health

© 2025 Anderson A et al. *JAMA Health Forum*.

Group and CVS Health exhibited the highest average number of threshold crossings per contract (2.13 and 2.00, respectively), indicating frequent movement across the 4-star bonus threshold. These organizations also had high proportions of contracts ever rated ≥4 stars (48% and 47%) and high rates of volatility (79% and 71% of contracts crossed the threshold at least once). In contrast, Centene and Molina had far lower average crossings (0.71 and 1.00, respectively) and a much smaller share of contracts ever reaching the bonus threshold (6% and 10% of years rated ≥4 stars). These patterns suggest that some organizations consistently hover around the threshold, experiencing frequent gains and losses in bonus status—while others remain stably below or above it. Kaiser Foundation Health Plan stands out with the highest average proportion of years rated ≥4 stars (90%) despite having fewer threshold crossings, reflecting sustained high performance with relatively low volatility. Overall, these findings underscore that the experience of bonus eligibility and rating volatility differs markedly across parent organizations, with implications for both payment incentives and contract stability.

© 2025 Anderson A et al. *JAMA Health Forum*.

eFigure. Distribution of Medicare Advantage Star Ratings, 2015–2025

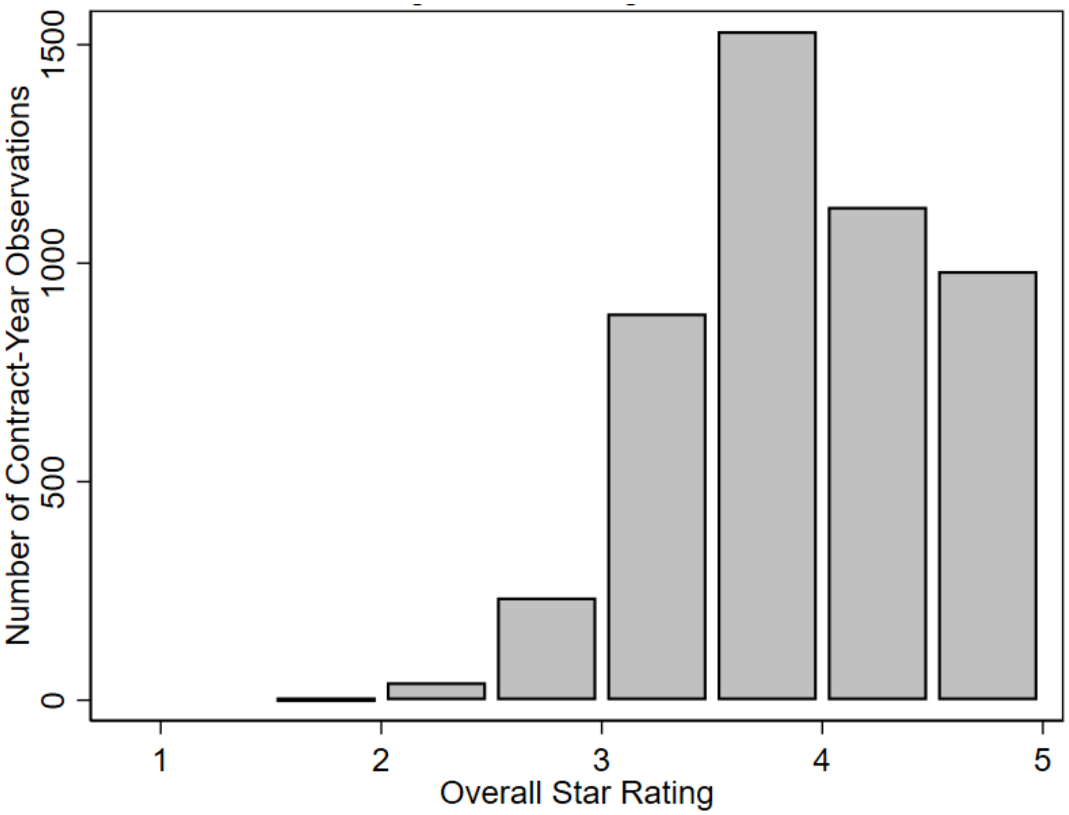

Appendix Figure A1. Histogram of Medicare Advantage contract-year star ratings from 2015–2025. Ratings are typically reported in half-star increments, and most contract-years cluster between 3.0 and 4.5 stars. A substantial proportion of observations fall near the 4.0 threshold, highlighting the potential for volatility in star ratings to influence bonus receipt.

© 2025 Anderson A et al. *JAMA Health Forum*.

eTable 1. Direction of Annual Star Rating Change by Year (Contract-Year Observations)

Year	Decreased	Increased	No Change	Total
2015	400	0	0	400
2016	136	107	131	374
2017	123	104	142	369
2018	142	91	160	393
2019	108	102	173	383
2020	113	138	158	409
2021	73	90	243	406
2022	89	261	129	479
2023	283	60	171	514
2024	244	126	181	551
2025	211	116	200	527

Note: This table summarizes the number of contract-year observations where a contract's star rating increased, decreased, or remained unchanged relative to the prior year. Observations from 2015 reflect the first observed year per contract and thus have no prior-year comparison. Across 4,805 contract-year observations from 2015–2025, 1,195 (25%) showed an increase, 1,922 (40%) a decrease, and 1,688 (35%) no change. Rating decreases were more common than increases, providing empirical context to concerns about asymmetric volatility in Medicare Advantage Star Ratings.

© 2025 Anderson A et al. *JAMA Health Forum*.

eTable 2. Robustness of Bonus Eligibility Models With 2-Year Minimum Observation Requirement (N = 659 Contracts)

	(1)<br>Proportion 4+ Years	(2)<br>Consecutive 4+ Years	(3)<br>Proportion 5-Star	(4)<br>Consecutive 5-Star
Bonus Entries	0.002**	-0.003**	-0.003***	-0.002***
	(0.001)	(0.001)	(0.001)	(0.000)
Bonus Exits	-0.000	-0.005***	-0.001**	-0.001**
	(0.001)	(0.001)	(0.001)	(0.000)
Mean Star Rating	0.574***	0.416***	0.160***	0.062***
	(0.016)	(0.017)	(0.016)	(0.010)
Log Total Enrollment	0.010*	0.006	-0.012***	-0.004**
	(0.005)	(0.005)	(0.003)	(0.002)
Mean SNP Enrollment (%)	0.009	-0.025	-0.015	-0.007
	(0.026)	(0.026)	(0.016)	(0.008)
Mean LIS Enrollment (%)	-0.001**	-0.001*	-0.000	-0.000
	(0.000)	(0.000)	(0.000)	(0.000)
Years Observed	-0.000	0.002***	0.000***	0.000***
	(0.000)	(0.000)	(0.000)	(0.000)
1876 Cost Plan	-0.012	-0.024	0.084	0.075
	(0.034)	(0.051)	(0.063)	(0.057)
Local CCP (Ref)	0.000	0.000	0.000	0.000
	(.)	(.)	(.)	(.)
MSA Plan	0.157	0.126	0.065	0.030
	(0.159)	(0.144)	(0.049)	(0.019)
PFFS Plan	-0.148***	-0.186***	-0.023**	-0.015**
	(0.030)	(0.036)	(0.011)	(0.007)
Regional CCP	-0.064**	-0.072***	-0.025***	-0.009**
	(0.025)	(0.023)	(0.010)	(0.004)
Constant	-1.776***	-1.357***	-0.390***	-0.160***
	(0.072)	(0.070)	(0.049)	(0.029)

© 2025 Anderson A et al. *JAMA Health Forum*.

Observations	659.000	659.000	659.000	659.000
R-squared	0.830	0.754	0.467	0.316

Data source**:** Centers for Medicare & Medicaid Services (CMS) Medicare Advantage Star Ratings Data Tables, 2015–2025.

Notes**:** This table presents linear regression models assessing the association between changes in bonus status and plan performance, using a broader sample of Medicare Advantage contracts with at least two years of data (compared to the main analysis which required ≥3 years). The exposures are the number of entries into and exits from bonus eligibility (defined as crossing the 4-star threshold in either direction) over the observed period. Outcomes include the proportion of years rated ≥4.0 ("Proportion 4+ Years"), proportion of consecutive years rated ≥4.0 ("Consecutive 4+ Years"), the proportion of years rated 5 stars ("Proportion 5-Star"), and the proportion of consecutive years rated 5 stars ("Consecutive 5-Star"). All models adjust for average star rating, log total enrollment, average SNP and LIS enrollment percentages, years observed, and plan type. Local CCP is the reference category. Standard errors are shown in parentheses.

© 2025 Anderson A et al. *JAMA Health Forum*.

eTable 3. Robustness of Bonus Eligibility Models With 4-Year Minimum Observation Requirement (N = 481 Contracts)

	(1) Proportion 4+ Years	(2) Consecutive 4+ Years	(3) Proportion 5-Star	(4) Consecutive 5-Star
Bonus Entries	0.002**	-0.003***	-0.003***	-0.002***
	(0.001)	(0.001)	(0.001)	(0.000)
Bonus Exits	-0.001	-0.005***	-0.001**	-0.001**
	(0.001)	(0.001)	(0.001)	(0.000)
Mean Star Rating	0.671***	0.566***	0.190***	0.086***
	(0.014)	(0.015)	(0.015)	(0.011)
Log Total Enrollment	0.009**	0.008*	-0.012***	-0.005**
	(0.005)	(0.005)	(0.004)	(0.002)
Mean SNP Enrollment (%)	0.015	0.001	-0.037**	-0.017
	(0.031)	(0.031)	(0.016)	(0.011)
Mean LIS Enrollment (%)	-0.001	-0.000	0.000	0.000
	(0.000)	(0.000)	(0.000)	(0.000)
Years Observed	-0.000	0.001***	0.000***	0.000***
	(0.000)	(0.000)	(0.000)	(0.000)
1876 Cost Plan	-0.044	-0.059	0.098	0.079
	(0.051)	(0.058)	(0.063)	(0.060)
Local CCP (Ref)	0.000	0.000	0.000	0.000
	(.)	(.)	(.)	(.)
MSA Plan	0.029	0.043	0.034	0.024**
	(0.060)	(0.091)	(0.024)	(0.010)
PFFS Plan	-0.099**	-0.132***	-0.005	-0.005
	(0.043)	(0.036)	(0.013)	(0.008)
Regional CCP	-0.041*	-0.053***	-0.014	-0.002
	(0.021)	(0.020)	(0.009)	(0.004)
Constant	-2.141***	-1.925***	-0.515***	-0.243***
	(0.065)	(0.068)	(0.052)	(0.036)

© 2025 Anderson A et al. *JAMA Health Forum*.

Observations	481.000	481.000	481.000	481.000
R-squared	0.901	0.870	0.567	0.394

Standard errors in parentheses
* $p < 0.10$, ** $p < 0.05$, *** $p < 0.01$

Data source: Centers for Medicare & Medicaid Services (CMS) Medicare Advantage Star Ratings Data Tables, 2015–2025.

Notes: This table presents linear regression models assessing the association between changes in bonus status and plan performance, using a more restrictive sample of Medicare Advantage contracts with at least four years of data (compared to the main analysis which required ≥3 years). The exposures are the number of entries into and exits from bonus eligibility (defined as crossing the 4-star threshold in either direction) over the observed period. Outcomes include the proportion of years rated ≥4.0 ("Proportion 4+ Years"), proportion of consecutive years rated ≥4.0 ("Consecutive 4+ Years"), the proportion of years rated 5 stars ("Proportion 5-Star"), and the proportion of consecutive years rated 5 stars ("Consecutive 5-Star"). All models adjust for average star rating, log total enrollment, average SNP and LIS enrollment percentages, years observed, and plan type. Local CCP is the reference category. Standard errors are shown in parentheses.

© 2025 Anderson A et al. *JAMA Health Forum*.

eTable 4. Robustness of Bonus Eligibility Models With 5-Year Minimum Observation Requirement (N = 402 Contracts)

	(1)<br>Proportion 4+ Years	(2)<br>Consecutive 4+ Years	(3)<br>Proportion 5-Star	(4)<br>Consecutive 5-Star
Bonus Entries	0.002**	-0.003***	-0.003***	-0.002***
	(0.001)	(0.001)	(0.001)	(0.000)
Bonus Exits	-0.001	-0.005***	-0.001*	-0.001*
	(0.001)	(0.001)	(0.001)	(0.000)
Mean Star Rating	0.685***	0.609***	0.207***	0.100***
	(0.014)	(0.013)	(0.015)	(0.011)
Log Total Enrollment	0.012**	0.010**	-0.012***	-0.007***
	(0.005)	(0.004)	(0.004)	(0.003)
Mean SNP Enrollment (%)	0.027	0.025	-0.057***	-0.039**
	(0.037)	(0.035)	(0.020)	(0.015)
Mean LIS Enrollment (%)	-0.001	-0.001	0.001**	0.000*
	(0.000)	(0.000)	(0.000)	(0.000)
Years Observed	-0.000	0.001***	0.000**	0.000**
	(0.000)	(0.000)	(0.000)	(0.000)
1876 Cost Plan	-0.096**	-0.106***	0.120*	0.092
	(0.041)	(0.037)	(0.072)	(0.072)
Local CCP (Ref)	0.000	0.000	0.000	0.000
	(.)	(.)	(.)	(.)
MSA Plan	0.039	0.061	0.038	0.029**
	(0.062)	(0.099)	(0.028)	(0.014)
PFFS Plan	-0.091**	-0.104***	0.004	0.001
	(0.044)	(0.040)	(0.013)	(0.007)
Regional CCP	-0.039*	-0.047**	-0.013	0.000
	(0.021)	(0.019)	(0.010)	(0.006)
Constant	-2.220***	-2.090***	-0.563***	-0.279***
	(0.061)	(0.060)	(0.055)	(0.039)

© 2025 Anderson A et al. *JAMA Health Forum*.

Observations	402.000	402.000	402.000	402.000
R-squared	0.916	0.905	0.619	0.454

Data source: Centers for Medicare & Medicaid Services (CMS) Medicare Advantage Star Ratings Data Tables, 2015–2025.

Notes: This table presents linear regression models assessing the association between changes in bonus status and plan performance, using a more restrictive sample of Medicare Advantage contracts with at least five years of data (compared to the main analysis which required ≥3 years). The exposures are the number of entries into and exits from bonus eligibility (defined as crossing the 4-star threshold in either direction) over the observed period. Outcomes include the proportion of years rated ≥4.0 ("Proportion 4+ Years"), proportion of consecutive years rated ≥4.0 ("Consecutive 4+ Years"), the proportion of years rated 5 stars ("Proportion 5-Star"), and the proportion of consecutive years rated 5 stars ("Consecutive 5-Star"). All models adjust for average star rating, log total enrollment, average SNP and LIS enrollment percentages, years observed, and plan type. Local CCP is the reference category. Standard errors are shown in parentheses.

© 2025 Anderson A et al. *JAMA Health Forum*.

eTable 5. Volatility and Bonus Eligibility by Parent Organization

Parent Organization	Number Of Contracts	Mean Enrollment	Mean Bonus Entries Per Contract	Mean Bonus Exits Per Contract	Mean Crossings Per Contract (Entries + Exits)	Mean Proportion of Years Rated ≥4	% Contracts Crossing Bonus Threshold
**United Health Group**	72.00	107189.01	1.03	1.10	2.13	0.48	0.79
**Centene Corporation**	52.00	20342.14	0.33	0.38	0.71	0.06	0.25
**Humana Inc**	48.00	114147.08	0.88	0.88	1.75	0.43	0.75
**Elevance Health, Inc.**	43.00	39127.11	0.72	0.84	1.56	0.30	0.53
**CVS Health**	38.00	71533.66	1.05	0.95	2.00	0.47	0.71
**The Cigna Group**	18.00	38199.52	0.72	0.78	1.50	0.34	0.67
**Molina Healthcare, Inc.**	13.00	8602.11	0.46	0.54	1.00	0.10	0.38
**Health Care Service Corporation**	10.00	13490.87	0.40	0.40	0.80	0.12	0.50
**Kaiser Foundation Health Plan, Inc.**	8.00	213540.34	0.50	0.75	1.25	0.90	0.75

Note: This table summarizes bonus eligibility volatility across the largest Medicare Advantage parent organizations, restricted to contracts with at least three years of data. Bonus entries and exits refer to transitions into or out of the 4-star quality bonus threshold. The table shows the number of contracts per parent organization, average enrollment, average number of bonus entries and exits per contract, average total crossings per contract, average proportion of years rated at or above 4 stars, and the share of contracts that ever crossed the 4-star threshold. Total entry and exit counts are included for context. Parent organization names have been standardized.
