# Supplementary material for: Fluctuating Star Ratings and Medicare Advantage Bonuses
Source: JAMA Health Forum. 2025 Oct 24;6(10):e254398. doi: 10.1001/jamahealthforum.2025.4398 (PMC12552924; doi:10.1001/jamahealthforum.2025.4398)
Supplement: Supplement 2. — Data sharing statement [file jamahealthforum-e254398-s002.pdf]

Data Sharing Statement

Anderson. Fluctuating Star Ratings and Medicare Advantage Bonuses. *JAMA Health Forum*. Published October 24, 2025. doi:10.1001/jamahealthforum.2025.4398

Data
Data available: Yes
Data types: Participant data with identifiers
How to access data: We will make it available in an online repository
When available: With publication

Supporting Documents
Document types: Statistical/analytic code
How to access documents: We will make it available in an online repository
When available: With publication

Additional Information
Who can access the data: publicly
Types of analyses: any purpose
Mechanisms of data availability: without investigator support
